# Genome-wide association study reveals a novel tuberculosis susceptibility locus in multiple East Asian and European populations

**DOI:** 10.1186/s13073-026-01670-6

**Published:** 2026-05-27

**Authors:** Xuling Chang, Zheng Li, Phan Vuong Khac Thai, Dang Thi Minh Ha, Nguyen Thuy Thuong Thuong, Denise Wee, Alya Sufiyah Binte Mohamed Subhan, Matthew Silcocks, Cynthia Bin Eng Chee, Nguyen Thi Quynh Nhu, Chew-Kiat Heng, Yik Ying Teo, Amit Singhal, Stefan H Oehlers, Jian-Min Yuan, Woon-Puay Koh, Maxine Caws, Chiea Chuen Khor, Rajkumar Dorajoo, Sarah J Dunstan

**Affiliations:** 1 Department of Infectious Diseases, University of Melbourne at the Peter Doherty Institute for Infection and Immunity, 792 Elizabeth St, Melbourne, Parkville, VIC 3000 Australia; 2 Department of Paediatrics, Yong Loo Lin School of Medicine, National University of Singapore, Singapore, 119228 Singapore; 3 Khoo Teck Puat – National University Children’s Medical Institute, National University Health System, Singapore, 119074 Singapore; 4 Genome Institute of Singapore (GIS), Agency for Science, Technology and Research (A*STAR), Singapore, 138672 Singapore; 5 Pham Ngoc Thach Hospital, District 5, Ho Chi Minh City, Viet Nam; 6 Oxford University Clinical Research Unit, District 5, Ho Chi Minh City, Viet Nam; 7 Centre for Tropical Medicine, Nuffield Department of Clinical Medicine, Oxford University, Oxford, UK; 8 A*STAR Infectious Diseases Labs (A*STAR ID Labs), Agency for Science, Technology and Research (A*STAR), Singapore, 138648 Singapore; 9 Tuberculosis Control Unit, Tan Tock Seng Hospital, Singapore, Singapore; 10 Department of Statistics and Applied Probability, National University of Singapore, Singapore, Singapore; 11 Saw Swee Hock School of Public Health, National University of Singapore, Singapore, Singapore; 12 Singapore Immunology Network (SIgN), Agency for Science, Technology and Research (A*STAR), Singapore, 138684 Republic of Singapore; 13 Department of Medicine, UMass Chan Medical School, Worcester, MA 01605 United States of America; 14 Lee Kong Chian School of Medicine, Nanyang Technological University (NTU), Singapore, 308232 Republic of Singapore; 15 Cancer Epidemiology and Prevention Program, University of Pittsburgh Medical Centre (UPMC) Hillman Cancer Center, Pittsburgh, PA 15232 USA; 16 Department of Epidemiology, School of Public Health, University of Pittsburgh, Pittsburgh, PA 15261 USA; 17 Healthy Longevity Translational Research Programme, Yong Loo Lin School of Medicine, National University of Singapore, Singapore, 117545 Singapore; 18 A*STAR Institute for Human Development and Potential, Singapore, Singapore; 19 Department of Clinical Sciences, Liverpool School of Tropical Medicine, Pembroke Place, Liverpool, L3 5QA UK; 20 Birat Nepal Medical Trust, 257 Lazimpat, Kathmandu, Nepal; 21 Singapore Eye Research Institute, Singapore, Singapore

**Keywords:** Genome-wide association study, Pulmonary tuberculosis, Infectious diseases, Underrepresented populations, Non-HLA genetic locus

## Abstract

**Background:**

Tuberculosis (TB) continues to be a leading cause of morbidity and mortality worldwide. Although numerous genome-wide association studies (GWAS) have explored TB susceptibility across various ethnic groups, multi-population replication of findings has been very limited, particularly outside the HLA region, and a significant portion of TB heritability remains unexplained.

**Methods:**

We conducted GWAS in the Singapore Chinese and Vietnamese, followed by a comprehensive meta-analysis incorporating 4 independent East Asian datasets (*N* = 11,841 cases; *N* = 197,373 controls). The transferability of any identified association was assessed using summary statistics from independent European populations. Potential candidate genes were prioritized using gene-based association testing and integrative bioinformatic database mining, followed by functional validation through assessment of *Mycobacterium marinum* (*M.marinum*) infection burden in CRISPR-Cas9-edited zebrafish embryos.

**Results:**

We identified a novel susceptibility locus for pulmonary TB (PTB) at 22q12.2 in East Asians [rs6006426, OR (95%Cl) = 1.097(1.066, 1.130), *P*_*meta*_=3.31 × 10^− 10^]. The association was further validated in Europeans [OR (95%Cl) = 1.101(1.002, 1.211), *P* = 0.046] and was strengthened in the combined meta-analysis including a total of 12,736 PTB cases and 673,864 controls [OR (95%Cl) = 1.098 (1.068, 1.129); *P*_*meta*_=4.33 × 10^− 11^]. Gene-based association test identified Oncostatin M (*OSM*) to be significantly associated with PTB (ZSTAT = 5.013; *P* = 2.68 × 10^− 7^; *P*_*adj*_=0.005). The lead SNP rs6006426 affected Splicing factor 3a subunit 1 (*SF3A1*) expression in various immune cells (*P* from 0.003 to 6.17 × 10^− 18^) and *OSM* expression in monocytes post lipopolysaccharide stimulation (*P* = 5.57 × 10^− 4^) as reported in the eQTL Catalogue. CRISPR-Cas9 edited zebrafish embryos with *osm* depletion resulted in decreased burden of *M.marinum* in infected embryos (*P* = 0.047).

**Conclusions:**

Our findings offer novel insights into the genetic factors underlying TB and reveals new avenues for understanding its etiology.

**Supplementary Information:**

The online version contains supplementary material available at 10.1186/s13073-026-01670-6.

## Background

Tuberculosis (TB), an infectious disease caused by *Mycobacterium tuberculosis* (*Mtb*), poses a significant challenge to global health and is a major contributor to the rising global burden of antimicrobial resistance [1]. The World Health Organisation (WHO) 2025 global TB report indicates an estimated 10.7 million people fell ill with TB and 1.23 million died from the disease globally in 2024. TB predominantly affects individuals in low- and middle-income countries, with the highest burden observed in Southeast Asia, the Western Pacific, and African regions [1]. Despite recent advances in treatment and diagnosis, the challenge to optimally disrupt transmission and ensure positive outcomes for all those infected remains. This enduring challenge is primarily attributed to several factors, such as reduced efficacy of the current vaccine, lengthy and complex multi-drug treatment, and the rising incidence of multidrug-resistant (MDR) and extensively drug-resistant (XDR) TB [2]. Deeper understanding of TB etiology is crucial for identifying those at high risk and for development of effective vaccines and targeted treatments, including host-directed therapies, to control this global health threat.

TB susceptibility is influenced by a complex interplay of genetic and environmental factors [3, 4]. Twin studies and mouse models demonstrate a strong host genetic influence on TB susceptibility [5, 6] with heritability estimated > 50% [7]. Since the advent of genome-wide association studies (GWAS) in 2005, significant strides in uncovering genetic contributions to diseases have been made, with the focus predominantly on non-communicable diseases in European (EUR) populations. Until September 2024, only 5,722 (0.84%) out of 681,210 associations recorded in the GWAS catalog [8], are infectious disease related. Additionally, the GWAS diversity monitor shows that 94.49% of GWAS participants are European [9]. Therefore, comparatively, large-scale human TB GWAS has been neglected due to the disease being over-represented in resource-limited countries, particularly within populations that are under-represented in human genomics research. Despite this, research investigating TB susceptibility via GWAS has been conducted in populations from Africa [10, 11], Russia [12], Iceland [13] and Asia [14–18]. However, these studies have not fully accounted for the genetic risk and have shown considerable location or ethnicity specific genetic associations, with minimal replication across populations [12, 13, 19].

Here we performed GWAS and meta-analysis to identify pulmonary TB (PTB) associated genetic variants shared among East-Asian populations. We first evaluated PTB case-control datasets from Singapore and Vietnam. To increase statistical power and confidence, we incorporated public data from Han Chinese [15] and Japanese [16], into the meta-analysis. We also evaluated the transferability of the association in a combined European dataset, from the UK biobank (UKB) and FinnGen [16]. We identified a novel locus at chromosome 22q12.2 and prioritised Splicing factor 3a subunit 1 (*SF3A1*) and Oncostatin M (*OSM*) as candidate genes underlying the association by querying published TB transcriptomic and proteomic data alongside expression quantitative trait loci (eQTL) from the eQTL Catalogue (https://www.ebi.ac.uk/eqtl/) [20]. Functional assessment of *osm* in the zebrafish-*Mycobacterium marinum* (*M.marinum*) infection model revealed a reduced burden of bacterial infection in the *osm* knockdown crispants, suggesting a protective role for *osm* suppression during infection.

## Methods

### Study samples

The Singapore Chinese participants from the Hokkien and Cantonese dialect groups were enrolled in the SCHS, a prospective population-based cohort study of 63,257 participants (27,959 men and 35,298 women) aged 45 to 74 recruited between April 1993 and December 1998 to investigate genetic and environmental determinants of cancer and other chronic diseases in Singapore [21]. Biospecimens were collected from a random 3% subset starting in 1994 and later expanded to all consenting participants (around half of the cohort). PTB cases were ascertained via linkage with the National Tuberculosis Notification Registry. Diagnosis is predominantly driven by passive case detection, when patients present with symptoms, such as persistent cough, blood-stained sputum, fever, chills, and night sweats. Cases were diagnosed by positive sputum smear and confirmed by microbiological culture of *Mtb*. Notifications primarily originate from public hospitals and the Tuberculosis Control Unit, supplemented by electronic records from the two Mycobacterial laboratories in Singapore, ensuring comprehensive data capture in the National Tuberculosis Notification Registry. Controls were SCHS participants with complete genotype and phenotype data who had no history of PTB.

Blood samples were collected from PTB patients from Ho Chi Minh City (HCMC), Vietnam recruited as part of a larger clinical study [22]. Briefly, 2,091 newly diagnosed PTB patients attending Pham Ngoc Thach Hospital outpatients department or one of 8 District TB Units (DTUs) in HCMC were recruited between December 2008 and July 2011. Patients sampled for the genetics study were 18 years or older, HIV negative, had no previous history of TB treatment and were sputum smear positive. DNA was extracted using Qiagen Blood Midi kits (Qiagen) and 1,650 underwent whole genome genotyping at the Genome Institute of Singapore. Vietnamese Kinh population controls are otherwise healthy adults with primary angle closure glaucoma who have been previously described [23].

SCHS was approved by the Institutional Review Board (IRB) at the National University of Singapore. The study involving Vietnamese was approved by the IRBs of the Hospital for Tropical Diseases HCMC Vietnam, Pham Ngoc Thach Hospital for Tuberculosis and Lung Disease HCMC Vietnam, Health Services of HCMC Vietnam, the Oxford Tropical Research Ethics Committee, Oxford University UK and the University of Melbourne Human Research Ethics Committee, Melbourne Australia (ID 21973). Written informed consent was obtained from all study participants for both studies. All procedures involving human participants were conducted in accordance with the principles of the Helsinki Declaration.

Summary statistics for the Han Chinese samples were obtained from Zheng et al., 2018 (10.6084/m9.figshare.7006310) [15]. PTB cases were diagnosed based on (1) positive sputum culture for *M.tb*; (2) presence of acid-fast bacilli in sputum smear, and (3) clinical presentation and radiological signs (such as X-ray or computed tomography scan). All TB diagnoses were ultimately confirmed by culture of *M.tb* from sputum, and patients with extrapulmonary TB were excluded. Controls were healthy individuals recruited from the same hospitals as the cases and were screened for a history of TB and a family history of TB. Participants with clinical signs or symptoms, chest X-ray findings suggestive of active TB, a history of TB, or prior anti-TB treatment were excluded. The *M.tb* infection status of these controls was unknown.

Summary statistics for Biobank Japan and the European populations were obtained from Sakaue et al. 2021 (https://humandbs.dbcls.jp/en/hum0197-v3-220) [16]. PTB cases in Biobank Japan were identified by curating past medical history records and text-mining was performed to retrieve disease records from the free-format electronic medical records using the ICD-10 code A15 (refers to respiratory TB, bacteriologically and histologically confirmed). Controls were participants without a PTB diagnosis or related conditions. Case harmonization across Biobank Japan, UK Biobank, and FinnGen was performed using phecode 010, as described in [16].

### Genotyping and imputation

In SCHS, 27,308 DNA samples were whole genome genotyped using the Illumina Global Screening Array (GSA). An additional 2,161 independent subjects from the SCHS CAD-nested case-control study were genotyped on Illumina HumanOmniZhonghua Bead Chip. Comprehensive information regarding genotyping and quality control (QC) protocols has been previously published [24]. In brief, samples with a call-rate below 95% or heterozygosity values exceeding ± 3 standard deviations were excluded. Pairwise identity-by-state (IBS) analysis was performed to identify cryptically related individuals, and for each related pair, the sample with the lower call-rate was removed. PCA was conducted using both 1000 Genomes Project reference populations and the SCHS cohort to detect potential outliers relative to the reported ethnicities. After QC, SNP alleles were standardized to the forward strand and mapped to the hg38 reference genome. Minimac4 (version 1.0.0) [25] was employed to impute additional autosomal SNPs using a local population-specific reference panels composed of 9,770 whole-genome sequences of local Singaporean population samples obtained from the SG10K initiative (SG10K Health) [26] on the Research Assets Provisioning and Tracking Online Repository (RAPTOR) [27].

The Vietnamese samples were genotyped using either the Infinium OmniExpress-24 or OmniExpress-12 array. 1,650 PTB cases were combined with 1,357 Vietnamese kinh population controls. Samples with call-rate < 95% or extreme heterozygosity (beyond ± 3 standard deviations, *N* = 47) were excluded. IBS analyses identified first and second-degree related samples, with lower call-rate sample from each detected pair removed (*N* = 83). PCA with 1000 Genomes Projects reference populations and within the Vietnamese samples identified and removed 12 ethnicity outliers. For SNP QC (Additional file 1: Table S1), monomorphic or rare SNPs (MAF < 1.0%), those with call-rates < 95.0% (*N* = 112,360), sex chromosome SNPs, SNPs shown different missingness (*N* = 7,088), and those displaying gross Hardy-Weinberg equilibrium (HWE) deviation (*P* < 10^− 6^, *N* = 13,601) were removed. SNPs were coded to the forward strand and mapped to hg19. IMPUTE v2 [28] was used to mutually impute variants with cosmopolitan 1000 Genomes haplotypes as reference panel (Phase 3) [29]. SNPs with impute information score < 0.6, MAF < 1.0% or non-biallelic SNPs were excluded from subsequent analyses. In both datasets, the PCA plots show cases and controls distributed without distinct separation, suggesting no phenotype-specific clustering (Additional file 2: Fig S1).

### Statistical analysis

Single-variant association tests were performed using genotype dosage data with an R package SAIGE (v1.3.0) [30] for the SCHS and the Vietnamese dataset. SAIGE accounts for sample relatedness and manages case-control imbalance of binary traits. Genome-wide significance threshold was set at 5×10^− 8^. To assess potential inflation in the study results, the genomic inflation factor (λ) was calculated. For each cohort, the first three principal components were included as covariates to account for population stratification based on inspection of λ (λ = 1.007 for the SCHS and 0.985 for the Vietnamese) and QQ plots, indicating adequate control of population structure.

For the meta-analysis, publicly available summary statistics from PTB GWAS in East Asian populations [15, 16] were obtained and combined with the SCHS and Vietnamese GWAS results. Prior to meta-analysis, all datasets were harmonized to hg19 using the UCSC LiftOver tool [31]. Only SNPs present in all datasets and passing QC in the original studies were included, resulting in 4,582,578 variants for analysis. Detailed information for each individual dataset included in the meta-analysis is provided in Additional file 1: Table S2. An inverse variance-weighted meta-analysis was employed using META (v1.7) [32], assuming a fixed effects model. Heterogeneity among the combined data was evaluated using Cochran’s Q [33] and a *P*-value (*P*_heterogeneity_) < 0.05 was determined to be significantly heterogeneous.

### Functional mapping and annotation

Lead SNPs discovered were functionally annotated using the SNP2GENE function in FUMA (v1.6.1) [34]. SNPs in Linkage disequilibrium (LD; r^2^ > 0.6 in 1000G ASN panel) with sentinel SNPs were identified. Genes located within a 10 kb region flanking each lead SNP were mapped as regional genes at the locus of interest. FUMA performs MAGMA gene analysis (v1.08) [35] using the default SNP-wide mean model and 1000G EAS population as a reference panel. Previously published research on TB transcriptomics and proteomics was explored to identify TB candidate genes [36–39]. Differentially expressed genes in monocytes between active PTB cases and controls (GSE126614) were also analysed in a mixed ethnicity population [40]. Cell-type specific eQTL data from eQTL Catalogue [41] was queried; the significance of eQTLs was assessed based on the number of potential candidate genes under investigation (*P* = 0.050/7 = 0.007).

### Knockdown of *sf3a1* and *osm* in zebrafish embryos and analysis of *M. marinum* infection burden

Four guide RNAs per gene target were prepared using a common scaffold oligo and in vitro transcription (New England Biolabs, Additional file 1: Table S3) [42]. Injection solutions were contained 1 µl phenol red dye (Sigma), 2 µl 500 ng/µl pooled guides, and 2 µl 10 µM Cas9 (IDT DNA). Injections were performed into the yolk of 1–2 cells stage fertilized zebrafish eggs and embryos were reared at 28 °C with the addition of PTU at 1 day post fertilization (dpf). Embryos were infected with ~ 200 CFU fluorescent *M. marinum* at 2 dpf and imaged for bacterial burden quantification by fluorescent pixel count at 5 dpf [43]. Quantification was not carried out blinded. Bacterial burden was averaged for each experiment and compared across 4 replicates by paired *t*-test. Statistical testing was carried out by paired *t*-test generating a correlation coefficient of 0.954 and one-tailed *P* value of 0.023 indicating effective pairing (Graphpad Prism).

## Results

### PTB genome-wide association study

We performed PTB GWAS using datasets from Singapore [the Singapore Chinese Health Study (SCHS) 1,610 PTB cases/24,015 population controls; 7,141,960 (hg38) single nucleotide polymorphism (SNPs)] [21] and Vietnam [1,598 PTB cases/1,267 population controls; 7,490,205 (hg19) SNPs]. After PCA-adjustment, no significant inflation was observed (Additional file 2: Fig S2), indicating that potential population stratification effects were well-controlled. No associations surpassed genome-wide significance threshold of *P* < 5 × 10^− 8^ in individual analyses (Additional file 2: Fig S2).

**Fig. 1:**
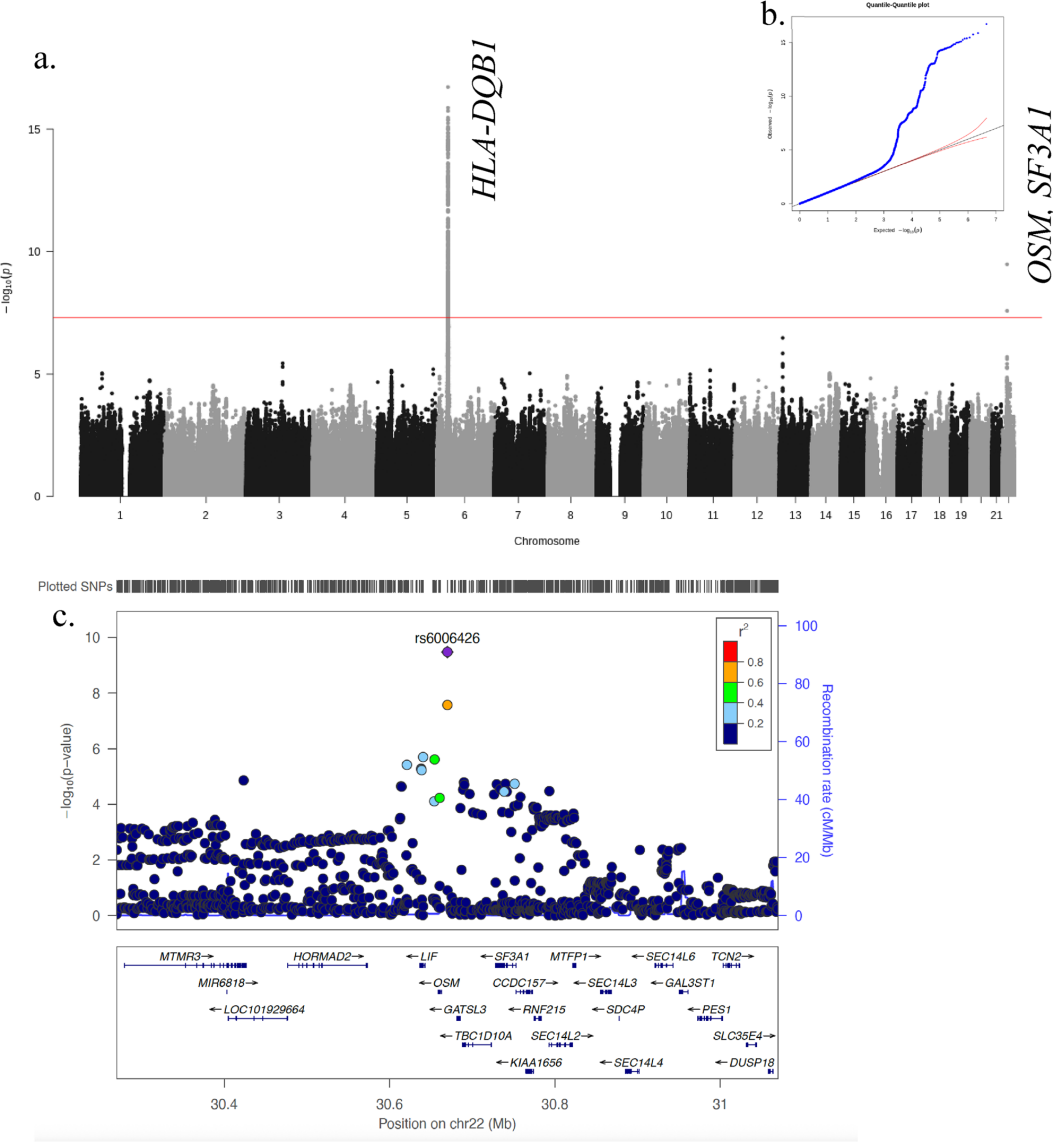
Meta-analysis for PTB in East Asia. **a** Manhattan plot of the meta-analysis of 4 Asian populations (11,841 cases/197,373 controls). Two hits at chromosome 6 and 22 were identified beyond the genome-wide significance threshold (*P* < 5 × 10^-8^, red line) **b** QQ-plot of observed compared to expected *P*-values indicated minimal inflation (*λ* = 1.028). **c** Regional SNP associations at 22q12.2 in the meta-analysis of 4 Asian populations. Lead SNP indicated as purple diamonds. LD (r^2^) data of SNPs based on ASN panels of 1000 Genome database. Plots plotted using LocusZoom (http://locuszoom.org/)

We next performed a meta-analysis incorporating all available PTB GWAS from East Asia. Published summary statistics from Han Chinese and Japanese (Biobank Japan) [15, 16] were merged with in-house GWAS data [4,582,578 overlapping SNPs] resulting in a combined dataset of 11,841 PTB cases and 197,373 controls. The East-Asian meta-analysis indicated minimal inflation (λ = 1.028) and identified two loci surpassing genome-wide significance (Fig. 1a and b), including the known *HLA-DQB1* locus [16]. A novel locus was identified at 22q12.2 [OR (95%Cl) = 1.097(1.066, 1.130), *P*_*meta*_=3.31 × 10^− 10^, Table 1; Fig. 1a, Additional file 2: Fig S3], which demonstrated significant associations across all included datasets (Table 1). The lead SNP, rs6006426, is an intergenic SNP 7,043 bp upstream of the transcription start site of the *OSM* as indicated by the Open Targets Platform (Fig. 1c) [44], with a minor allele frequency (MAF) of 37%-47% in East Asian (EAS) populations according to the 1000 Genome Project [29].

**Table 1:** Summary statistics of genome-wide associations identified in individual datasets and after meta-analysis for pulmonary tuberculosis

	SNP	chromosome	position	Other allele	Effect allele	Sample size (cases/controls)	OR (95%Cl)	*P*	*P*_heterogeneity_
meta-analysis EAS	rs6006426	22	30,669,883	G	A	11,841/197,373	1.097 (1.066, 1.130)	3.31 × 10^− 10^	0.079
SCHS						1,610/24,015	1.099 (1.018, 1.187)	0.016	
Vietnamese						1,598/1,267	1.169 (1.032, 1.324)	0.014	
Han Chinese						833/1,220	1.282 (1.120, 1.468)	3.16 × 10^− 4^	
Biobank Japan						7,800/170,871	1.082 (1.046, 1.119)	3.69 × 10^− 6^	
meta-analysis EAS + EUR						12,736/673,864	1.098 (1.068, 1.129)	4.33 × 10^− 11^	0.148
UKB+FinnGen						895/476,491	1.101 (1.002, 1.211)	0.046	

*OR* Odds ratio, *Cl* Confidence interval, *EAS* East Asian, *EUR* European, *SCHS* Singapore Chinese Health Study, *UKB* UK Biobank

Previous studies have reported that men have a higher risk of *M.tb* immunoreactivity than women, which is thought to contribute to the sex differences observed in global TB morbidity and mortality [45, 46]. In our in-house datasets from Singapore and Vietnam, sex was self-reported for the SCHS and Vietnamese cases, and inferred from genotyping data for the Vietnamese controls. Consistent with prior reports, we observed a higher proportion of males among PTB cases (Additional file 1: Table S2). To assess whether the association between rs6006426 and PTB could be confounded by sex imbalance, we repeated the analysis by including sex as an additional covariate in the SCHS and Vietnamese for which individual-level data were available. Adjustment for sex did not substantially alter the effect estimates, and the association between rs6006426 and PTB remained consistent in both the SCHS [OR (95%Cl) = 1.096 (1.014, 1.184), *P* = 0.021] and Vietnamese [OR (95%Cl) = 1.169 (1.021, 1.338), *P* = 0.023], indicating that the association is not driven by sex.

We assessed the transferability of the above association using summary statistics from European populations [16]. A significant association was observed [OR (95%Cl) = 1.101(1.002, 1.211), *P* = 0.046, Table 1], and the overall meta-analysis was further strengthened after combining data from EAS and EUR [OR (95%Cl) = 1.098(1.068, 1.129), *P*_*meta*_=4.33 × 10^− 11^, Table 1, Additional file 2: Fig S3].

We also examined our lead SNP rs6006426 in the latest multi-ancestry meta-analysis [47]. This variant showed significant associations with TB in both Asian [OR (95%Cl) = 1.069 (1.006, 1.137), *P*_*meta*_=0.033] and European population [OR (95%Cl) = 1.057 (1.004, 1.113), *P*_*meta*_=0.036], and a directionally consistent effect in African populations [OR (95%Cl) = 1.071 (0.991, 1.157), *P*_*meta*_=0.082], with an overall significant association [chi-square (χ²) = 11.95, *P*_*meta*_=0.008, Additional file 1: Table S4]. However, as the dataset in this latest multi-ancestry meta-analysis includes a subset of individuals from Biobank Japan, which overlaps with our cohort, we could not include it in our meta-analysis because there would be sample duplication.

### Functional mapping and annotation

**Fig. 2:**
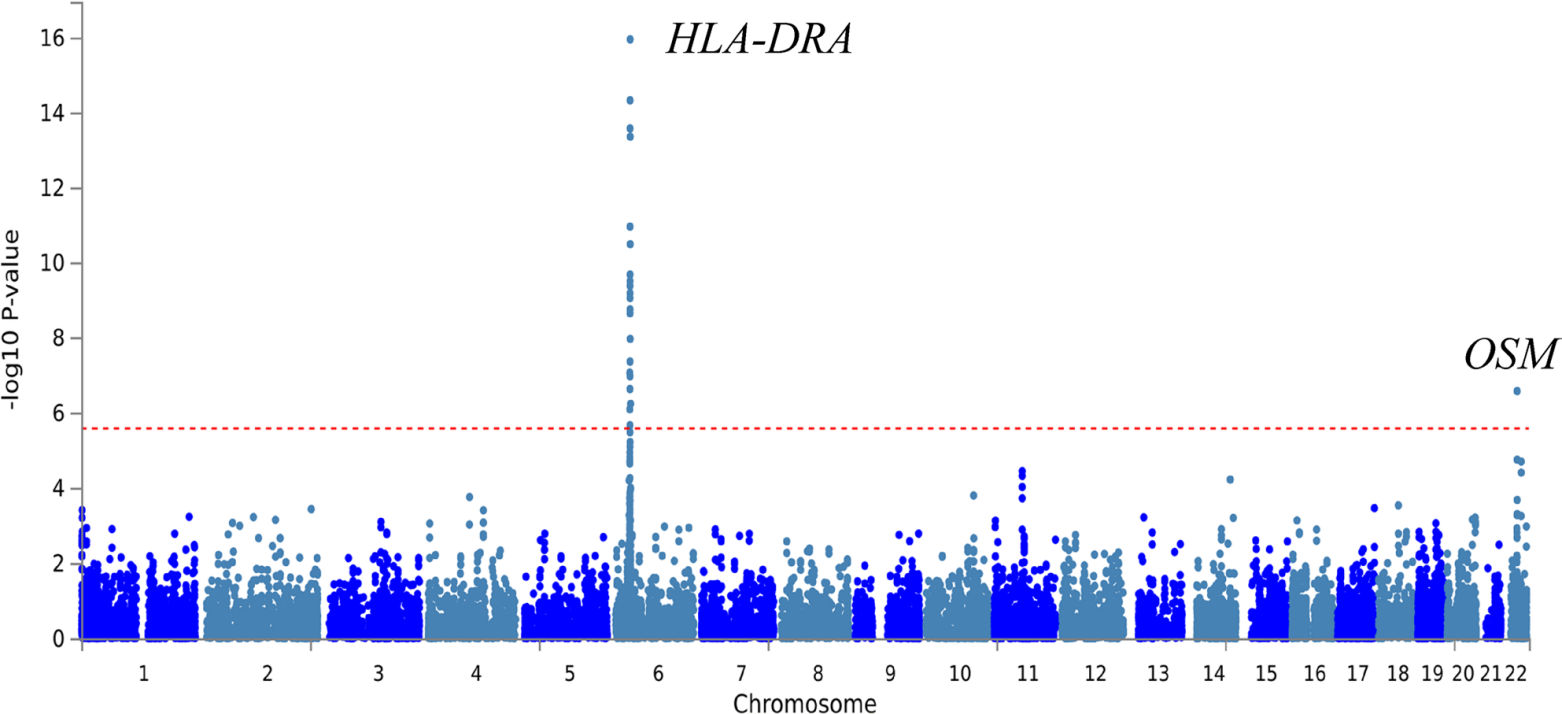
Manhattan plot of the gene-based test as computed by MAGMA using results from meta-analysis (11,841 cases/197,373 controls) as input. Input SNPs were mapped to 18,840 protein coding genes. Genome wide significance (red dashed line in the plot) was defined at *P* = 0.050/18,840 = 2.65 × 10^− 6^

Regional information for rs6006426 displayed using LocusZoom (http://locuszoom.org/) revealed that this locus is situated in a gene-rich area that encompasses multiple genes within ± 400 kb of the lead SNP (Fig. 1c). To prioritise genes potentially responsible for this association, we conducted gene-based test using MAGMA (v1.08), which uses a multiple regression approach to properly incorporate LD between markers and to detect multi-marker effects [35]. This was implemented in FUMA GWAS (Functional Mapping and Annotation of Genome Wide Association Studies) [34] using the East Asian meta-analysis data. In addition to the Major Histocompatibility Complex (MHC) region on chromosome 6, *OSM* was significantly associated with PTB (ZSTAT = 5.013; *P* = 2.68 × 10^− 7^; *P*_*adj*_=0.005) (Fig. 2, Additional file 1: Table S5).

We examined published TB transcriptomic and proteomic data to explore the potential functional link of candidate genes (within ± 400 kb of rs6006426) with TB [36–39]. In African and mixed populations, *OSM*, *TCN2*, and *THOC5* were differentially expressed in various TB phenotypic comparisons and *LIF* had higher protein abundance in those who progressed to active TB (Additional file 1: Table S6) [36–39]. To explore function of these genes in Asian populations, we assessed differential gene expression in monocytes of active PTB and healthy controls, from a previously published [40] mixed ancestry population of Singaporean Chinese, Malays, and Indians (20 PTB cases and 19 controls). *SF3A1*, *ASCC*,* LIF* and *MTMR3* showed differential expression, with the most significant, *SF3A1*, being downregulated in monocytes from PTB patients (Additional file 2: Fig S4). Together, these analyses highlighted seven candidate genes with potential functional relevance to TB pathogenesis. We then queried eQTL in immune cells from the eQTL Catalogue. rs6006426 significantly influences *SF3A1* expression in various immune cell regardless of exposure to viral, bacterial, or molecular stimuli (*P* ranging from 0.003 to 6.17 × 10^− 18^, Additional file 1: Table S7) [48–50]. The PTB risk increasing allele (allele A) was associated with reduced *SF3A1* expression. rs6006426 was not significantly associated with *OSM* expression in naive monocytes in most studies, however following stimulation with lipopolysaccharide (LPS) for 2 h, the PTB risk allele significantly decreased *OSM* expression (β=-0.305, *P* = 5.57 × 10^− 4^, Additional file 1: Table S7) [50]. The difference of *OSM* expression level among genotypes decreased with time and became less significant after 24 h (β=-0.089, *P* = 0.016, Additional file 1: Table S7) [50]. From these data, *SF3A1* and *OSM* emerged as plausible candidate genes.

### Function of *sf3a1* and *osm* in the zebrafish-*M.marinum* infection model

**Fig. 3:**
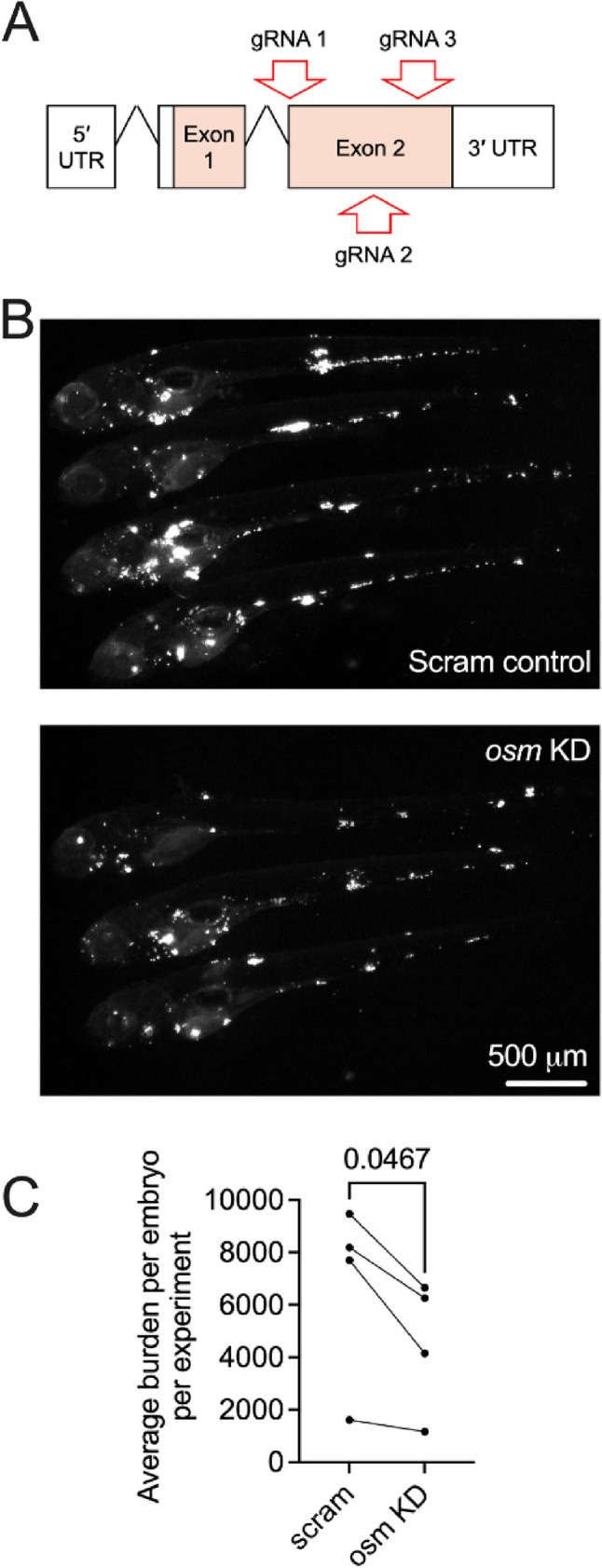
Functional analysis of *osm* in the zebrafish-*M. marinum* infection model. **a** Schematic of the zebrafish *osm* genomic locus with CRISPR-Cas9 gRNA target sites annotated; (**b**) Representative images of fluorescent *M. marinum* in *osm* knockdown zebrafish embryos; (**c**) Comparison of average *M. marinum* infection burden in *osm* knockdown zebrafish embryos, *n* = 4 experiments. Number of embryos used per experiment (control / knockdown / total): 33 / 39 / 72, 24 / 24 / 48, 14 / 16 / 30, and 33 / 33 / 66

Zebrafish are a valuable model for studying mycobacterial pathogenesis and treatment. Infection with *M. marinum*, the closest relative of the *Mtb* complex, mirrors many key aspects of human tuberculosis [51]. *SF3A1* and *OSM* were individually knocked down in zebrafish embryos and the infection burden of *M. marinum* was assessed. Using standard dose CRISPR-Cas9 injections to knock down *sf3a1*, we observed a higher *M. marinum* burden in the crispants compared to the scramble control (Additional file 2: Fig S5a). However, the survival rate of these crispants was low, and they showed noticeable embryo morphological abnormalities (Additional file 2: Fig S5b). Upon reducing the CRISPR-Cas9 injection dose to a level that did not elicit morphological abnormalities, the *M. marinum* burden became comparable (Additional file 2: Fig S5c). Therefore, we hypothesize that the initial infection phenotype was likely due to non-specific developmental defects consistent with the early broad expression pattern of *sf3a1* observed during development [52]. Targeting of *osm* by CRISPR-Cas9 mutagenesis was well tolerated by zebrafish embryos (Fig. 3a), and we observed a decreased burden of *M.marinum* in the *osm* knockdown crispants (Fig. 3b and c), suggesting a protective role for *osm* suppression during infection. The observed effect of *osm* knockdown in zebrafish is suggestive (*P* = 0.047) and warrants replication in independent experiments for a conclusive finding.

## Discussion

Genetic, environmental and socio-economic factors significantly contribute to TB susceptibility [3, 4]. Our comprehensive GWAS with the largest sample size to date in East Asians identified a novel PTB susceptibility locus. The consistent significant association across 5 independent datasets has been rarely observed in prior TB GWAS. The observed effect size is modest, which is consistent with findings from other complex traits where individual variants exert small effects [53]. As a result, huge sample sizes are normally required to detect statistically robust associations, which is a challenge for many infectious disease studies. While modest odds ratios suggest limited standalone translational utility, they highlight the polygenic nature of PTB and underscore the value of collaborative large-scale meta-analysis, while also revealing new avenues for biological insight. We identified *OSM* and *SF3A1* as potential candidate genes through functional mapping and annotation, neither of which has been reported in any genome-wide scaled analyses before. We showed *SF3A1* expression to be downregulated in monocytes from Singaporean PTB patients, and knocking down *osm* in a zebrafish model decreased the burden of *M. marinum* infection.

Previous GWAS have explored the genetic predisposition to TB in diverse populations [10–18]. These studies have yielded inconsistent results, which have been rarely replicable between studies. For instance, GWAS conducted in Ghanaian and Gambian identified 18q11.2 and 11p13 [10, 11], but only the 11p13 locus was replicated in Russia [12] and the South African study [19]. Additionally, the *ASAP1* gene was reported in a Russian cohort [12], but was not replicated in an Icelandic population [13]. In the most recent large-scale meta-analysis by the International Tuberculosis Host Genetics Consortium (ITHGC), which included 14,153 PTB cases and 19,536 controls across African, Asian and European populations, only one genome-wide significant locus was identified in the HLA region, and previously reported TB-associated variants were not replicated [47]. These inconsistencies could stem from various factors, such as differing LD structures in distinct populations or phenotypic heterogeneity in case or control ascertainment. As *Mtb* lineages display substantial geographic variation and have been suggested to have co-evolved with human populations [54], pathogen genomic variation might also underlie some of the challenges in replicating across populations [55]. These factors contribute to the increased complexity of conducting successful TB GWAS, in addition to performing GWAS in under-studied ethnicities that lack extensive reference genome panels. These findings underscore the importance of exercising caution when extrapolating GWAS results to different populations, highlighting the challenges of translating genetic associations across diverse ethnic groups. Conversely, our consistently replicated results show that combining datasets from genetically similar backgrounds enhances analytical power and facilitates discovery of novel TB loci.

The novel locus at 22q12.2 was supported by both single-variant and gene-based analyses. Multiple lines of evidence support *SF3A1* and *OSM* as the genes driving the association. *SF3A1* encodes a component of the splicing factor 3a (SF3A) protein complex, involved in pre-mRNA splicing as part of the spliceosome [56]. A previous study reported that the immune system responds to infections through different mechanisms, one of which is alternative splicing [57]. Dysregulation of, or mutations in, *SF3A1* may disrupt its role in pre-mRNA splicing, potentially affecting the immune response to bacterial infections. Inhibition of SF3A1 or SF3B1 increased production of a short form of MyD88 mRNA (MyD88_S_), a negative regulator of innate immunity through Toll-like receptor (TLR) signalling [58]. MyD88_S_ level is critical in determining the production of inflammatory cytokines in murine macrophages [59]. SF3A and SF3B mRNA splicing complexes function together in TLR signalling to regulate the production of MyD88_S_, and thereby control the extent of innate immune activation [58, 59]. Considering that innate immune cells are the initial line of defence against *Mtb* [60] and that *Mtb* can potentially be eradicated by the innate immune system before the onset of an adaptive immune response [61], the modulation of *SF3A1* expression is particularly significant. Our findings suggest that the A allele of rs6006426 may exhibit reduced *SF3A1* expression, potentially leading to higher MyD88_S_ mRNA level, which could limit the activation of the innate immune system, rendering those exposed and infected more susceptible to progression to active TB disease. A previous candidate gene study in the Han Chinese established a connection between *SF3A1* and TB [62]. Our sentinel SNP showed weak LD with the previously reported variants, rs2074733 (r^2^: 0.233 to 0.336) and rs10376 (r^2^: 0.056 to 0.099, EAS in 1000 Genomes Project East Asian). While rs2074733 was associated with PTB in 3 of our datasets, the direction of effect was inconsistent, potentially due to variations in case group composition. No association with rs10376 was found in our analysis (Additional file 1: Table S8). The functional impact of *sf3a1* could not be evaluated in the *M.marinum*-zebrafish infection model due to the non-specific developmental defects, and the potential essentiality of this gene. We propose further follow up studies are necessary using in vitro or monocyte/macrophage lineage-specific in vivo approaches to circumvent the role of *sf3a1* in embryonic development and reveal any TB-related phenotypes.

*OSM* is a secreted cytokine and growth regulator. eQTL data indicated that rs6006426 modulates *OSM* expression in monocytes after treatment with LPS, with the PTB risk allele significantly decreasing *OSM* expression. As LPS is a component of gram-negative bacterial cell walls that triggers TLR4 signalling [63], the eQTL effects might differ following stimulation of monocytes by *Mtb* ligands, or *Mtb* infection. A bioinformatics-based study reported *OSM* to be one of five upregulated genes predicting progression of latent tuberculosis infection (LTBI) to active TB in African and UK cohorts [64]. A mechanistic study using cell culture and mouse models found *Mtb*-induced OSM produced by leukocytes drives expression of matrix metalloproteinases MMP-1 and MMP-3, and suppression of matrix metalloproteinase inhibitors, by pulmonary fibroblasts [65]. OSM may therefore drive tissue destruction and facilitate dissemination of active TB and exacerbation of latent/subclinical TB. Although the PTB risk allele is associated with lower *OSM* expression in human monocytes, potentially increasing disease susceptibility, *osm* knockdown in zebrafish reduces mycobacterial burden. This apparent discrepancy likely reflects species differences, variations in infection models, or context-specific roles of *OSM* in immune regulation, including effects on granuloma formation and inflammatory responses. Nevertheless, these results indicate that *OSM* plays a role during bacterial infection. Further in vivo studies are required to decipher the TB context-dependent mechanism of allele-specific OSM-induced susceptibility and to link monocyte/macrophage production of OSM to granuloma matrix remodelling. The region identified in this large-scale meta-analysis may represent a shared regulatory area for both *OSM* and *SF3A1*, which together influence PTB risk. This mechanism might work similarly to how JAZF1 and TSPAN8 interact in diabetes [66]. Further functional experiments are necessary to uncover the exact mechanism involved.

The primary strength of our study lies in the integrative meta-analysis of multiple East Asian populations, significantly augmenting the sample size and enhancing statistical power. However, the use of the general population as control subjects is a limitation. We combined individuals with a range of TB exposure statuses, including those never exposed to *Mtb*, those exposed but not infected, and those who have been exposed and *Mtb* infected but remain asymptomatic. This heterogeneity within the control group potentially leads to an underestimation of the true effect size of the association but is unlikely to result in false positive findings, particularly by including individuals who are susceptible but have never been exposed or have not yet progressed to active disease. In the latest multi-ancestry meta-analysis [47], it has been shown that variation in prior exposure among controls can mask host genetic effects, with the largest impact observed for variants with moderate associations, while the most significant associations remained largely unchanged.

## Conclusions

Our meta-analysis identified 22q12.2 as a novel susceptibility locus for PTB in both East Asian and European populations. Although multiple lines of functional evidence suggest *SF3A1* and *OSM* as plausible candidate genes, the actual causal gene has not yet been fully determined. Our findings reveal new avenues for research, and further studies are necessary to elucidate the underlying mechanisms.

## Supplementary Information

Additional file 1: Supplementary Table S1-S8.

Additional file 2: Supplementary Fig S1-S5.

## Data Availability

Summary statistics for the pulmonary tuberculosis (PTB) GWAS in Singaporean Chinese and Vietnamese populations, as well as the East Asian meta-analysis generated in this study, are publicly available in figshare at: 10.6084/m9.figshare.30909305 [67].

## Abbreviations

DTUs: District TB Units
EAS: East Asian
EUR: European
eQTL: Expression quantitative trait loci
XDR: Extensively drug-resistant
FUMA GWAS: Functional Mapping and Annotation of Genome-Wide Association Studies
GWAS: Genome-wide association studies
GSA: Global Screening Array
HWE: Hardy–Weinberg equilibrium
HCMC: Ho Chi Minh City
IBS: Identity-by-state
IRB: Institutional Review Board
LTBI: Latent tuberculosis infection
LD: Linkage disequilibrium
LPS: Lipopolysaccharide
MHC: Major Histocompatibility Complex
MAF: Minor allele frequency
MDR: Multidrug-resistant
M.marinum: Mycobacterium marinum
Mtb: Mycobacterium tuberculosis
PTB: Pulmonary Tuberculosis
OSM: Oncostatin M
QC: Quality control
MyD88_S_: Short form of MyD88 mRNA
SCHS: Singapore Chinese Health Study
SNP: Single nucleotide polymorphism
SF3A: Splicing factor 3a
SF3A1: Splicing factor 3a subunit 1
TLR: Toll-like receptor
TB: Tuberculosis
UKB: UK biobank
WHO: World Health Organisation
