# Supplementary material for: Genome-wide association study reveals a novel tuberculosis susceptibility locus in multiple East Asian and European populations
Source: Genome Med. 2026 May 27;18:71. doi: 10.1186/s13073-026-01670-6 (PMC13214086; doi:10.1186/s13073-026-01670-6)
Supplement: Supplementary file 2 — Additional file 2: Supplementary Fig S1-S5. [file 13073_2026_1670_MOESM2_ESM.pptx]

## Slide 1
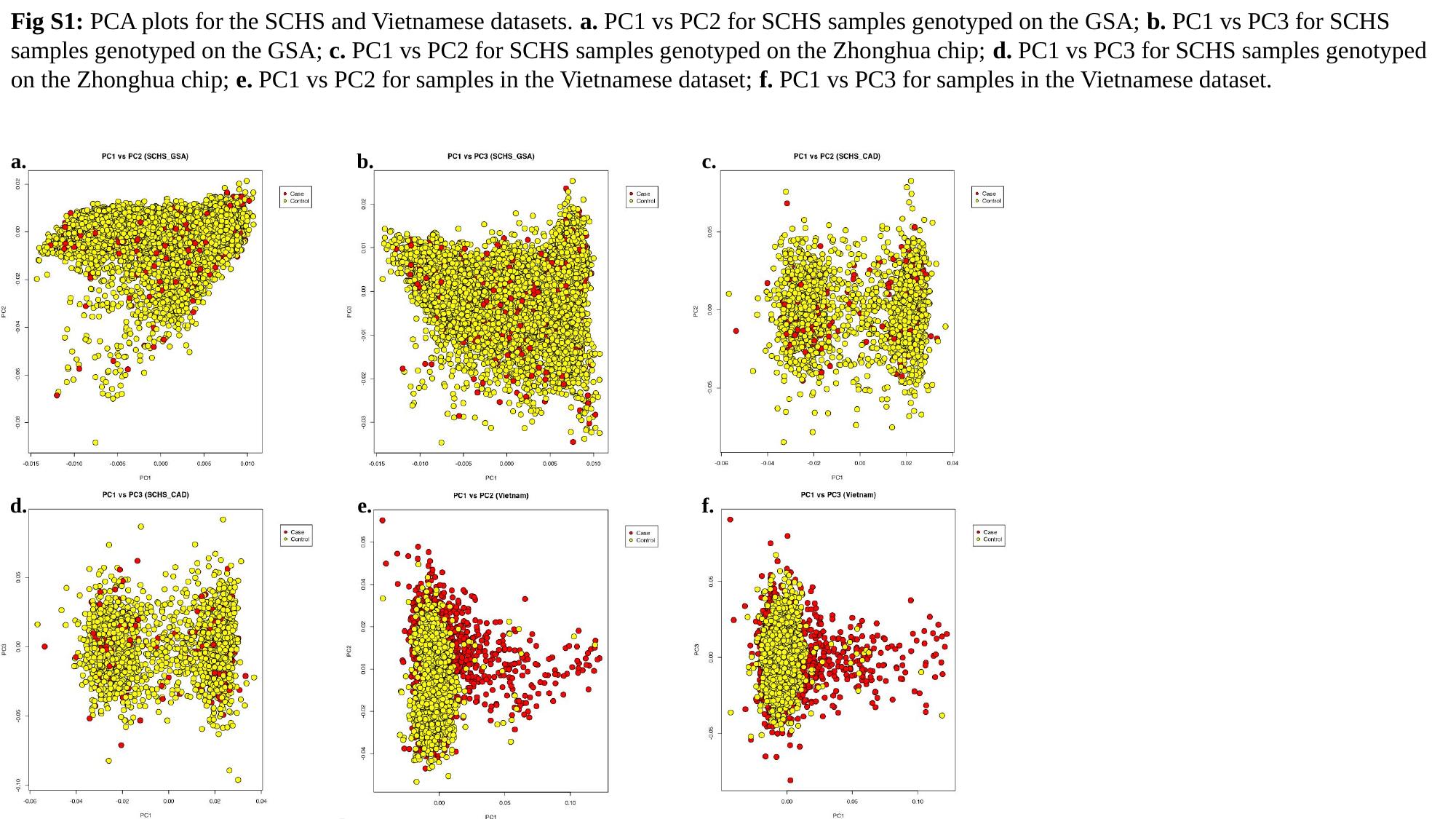

Fig S1: PCA plots for the SCHS and Vietnamese datasets. a. PC1 vs PC2 for SCHS samples genotyped on the GSA; b. PC1 vs PC3 for SCHS samples genotyped on the GSA; c. PC1 vs PC2 for SCHS samples genotyped on the Zhonghua chip; d. PC1 vs PC3 for SCHS samples genotyped on the Zhonghua chip; e. PC1 vs PC2 for samples in the Vietnamese dataset; f. PC1 vs PC3 for samples in the Vietnamese dataset.
a.
b.
c.
f.
e.
d.

## Slide 2
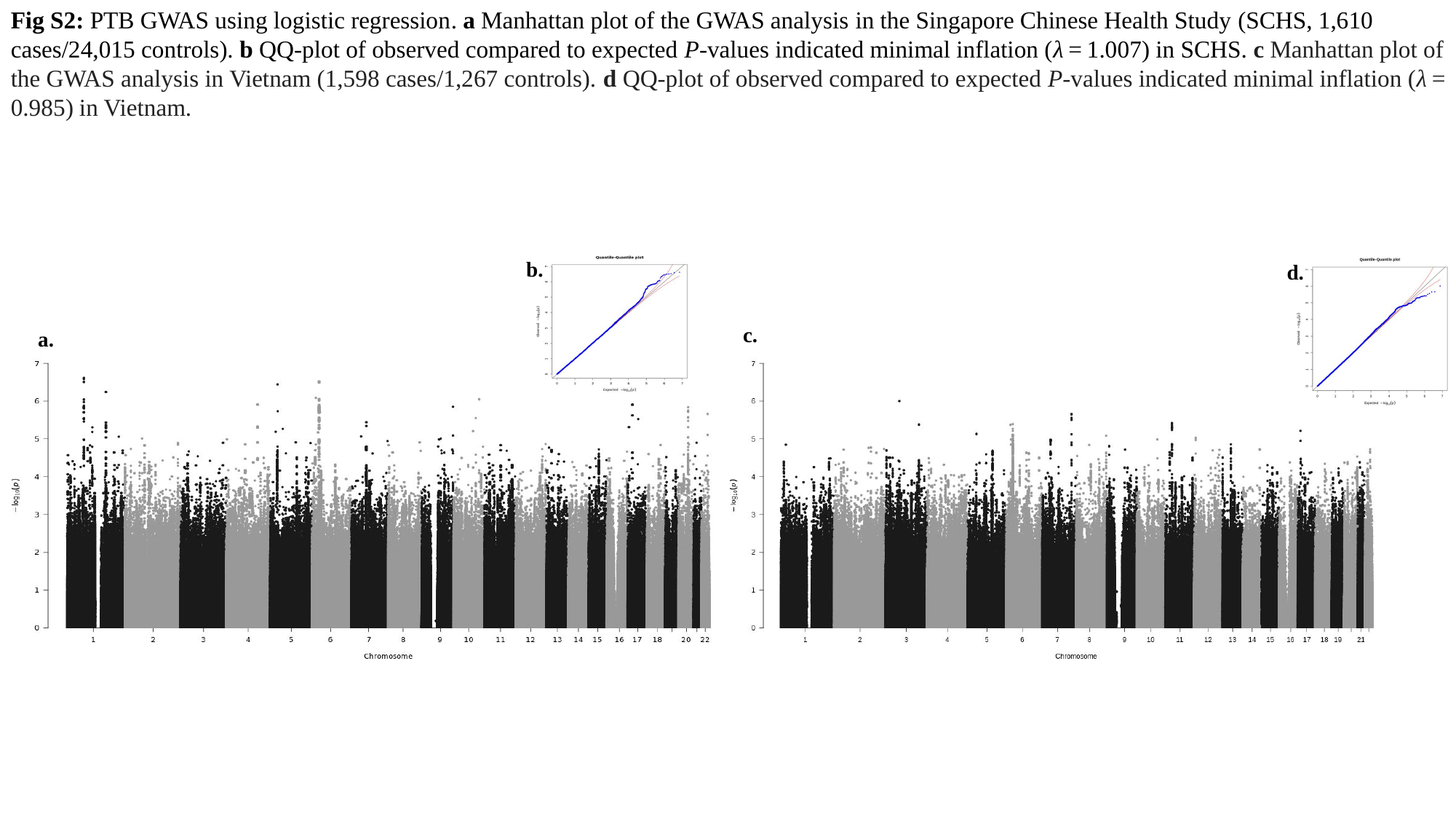

Fig S2: PTB GWAS using logistic regression. a Manhattan plot of the GWAS analysis in the Singapore Chinese Health Study (SCHS, 1,610 cases/24,015 controls). b QQ-plot of observed compared to expected P-values indicated minimal inflation (λ = 1.007) in SCHS. c Manhattan plot of the GWAS analysis in Vietnam (1,598 cases/1,267 controls). d QQ-plot of observed compared to expected P-values indicated minimal inflation (λ = 0.985) in Vietnam.
b.
a.
d.
c.

## Slide 3
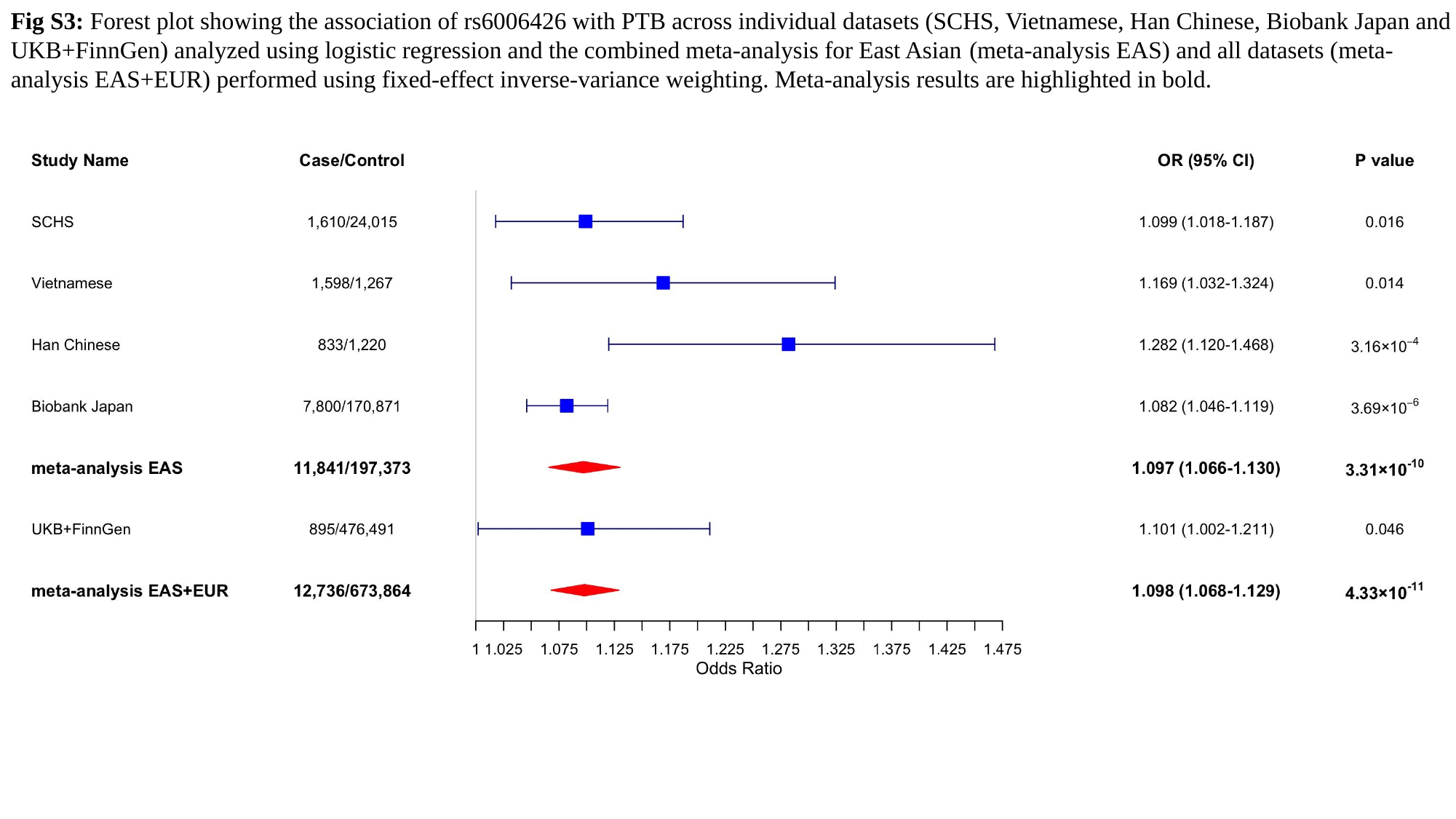

Fig S3: Forest plot showing the association of rs6006426 with PTB across individual datasets (SCHS, Vietnamese, Han Chinese, Biobank Japan and UKB+FinnGen) analyzed using logistic regression and the combined meta-analysis for East Asian (meta-analysis EAS) and all datasets (meta-analysis EAS+EUR) performed using fixed-effect inverse-variance weighting. Meta-analysis results are highlighted in bold.

## Slide 4
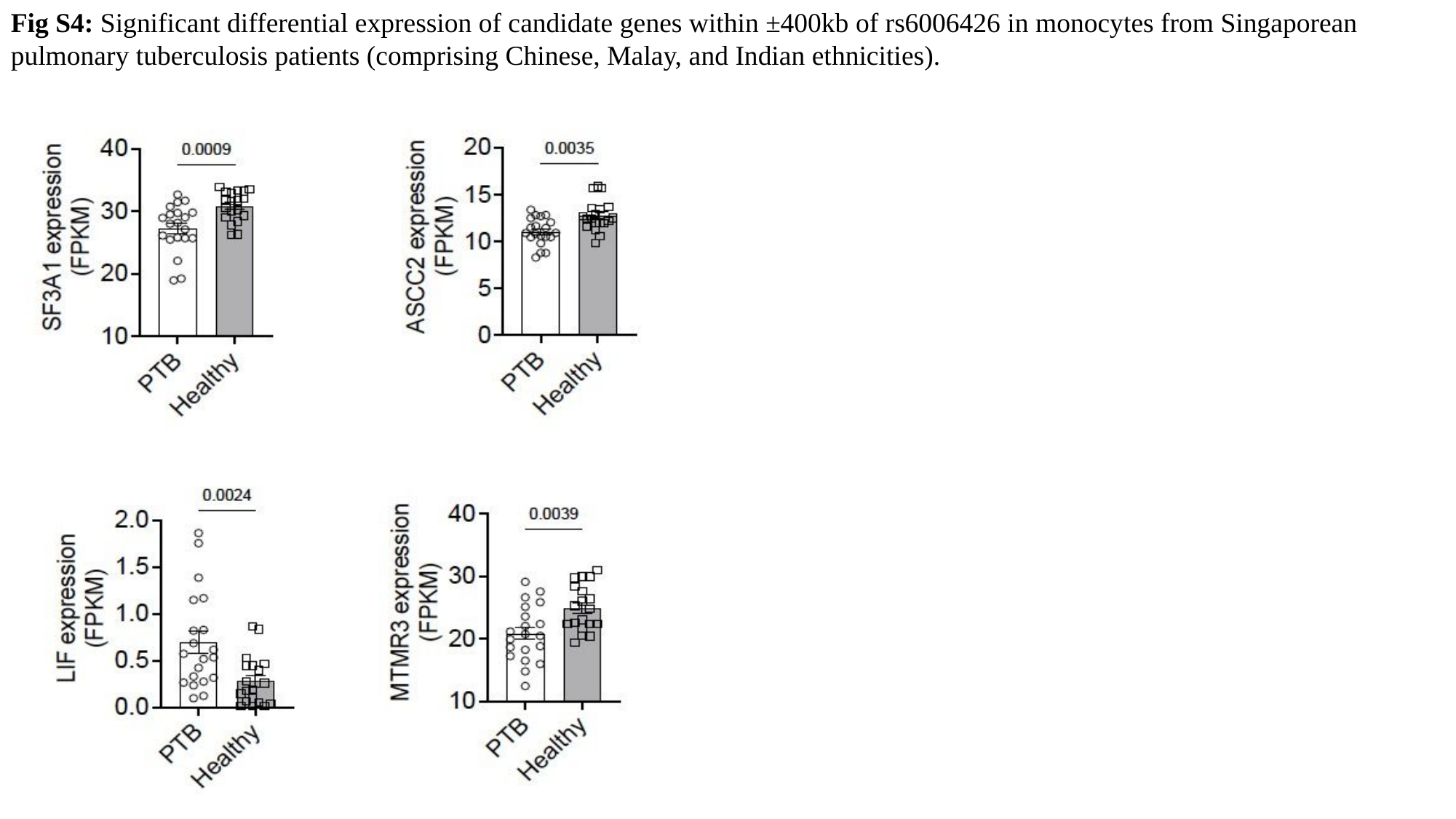

Fig S4: Significant differential expression of candidate genes within ±400kb of rs6006426 in monocytes from Singaporean pulmonary tuberculosis patients (comprising Chinese, Malay, and Indian ethnicities).

## Slide 5
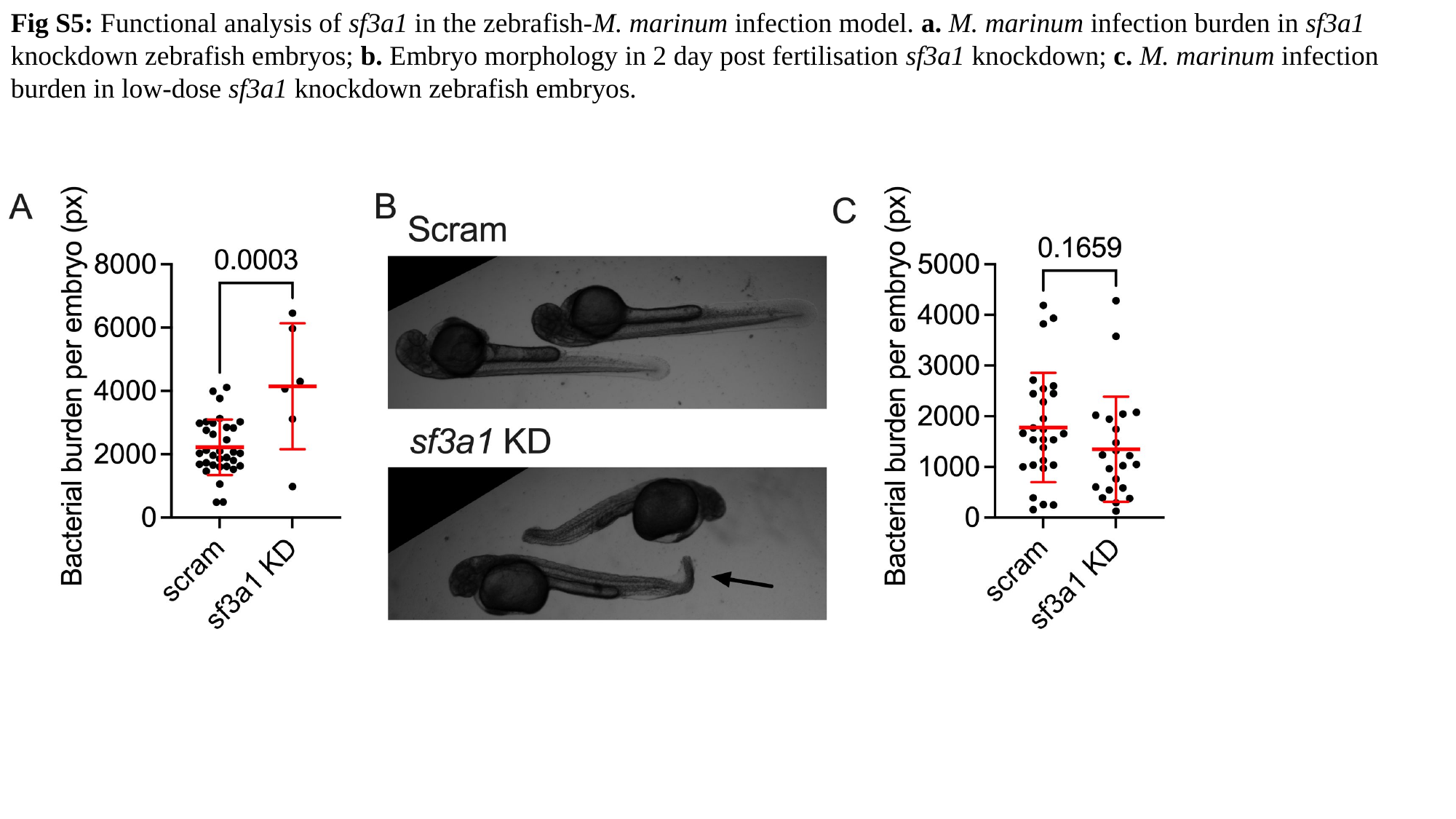

Fig S5: Functional analysis of sf3a1 in the zebrafish-M. marinum infection model. a. M. marinum infection burden in sf3a1 knockdown zebrafish embryos; b. Embryo morphology in 2 day post fertilisation sf3a1 knockdown; c. M. marinum infection burden in low-dose sf3a1 knockdown zebrafish embryos.
